# Retraction Note: Epigenetic modification of ferroptosis by non-coding RNAs in cancer drug resistance

**DOI:** 10.1186/s12943-026-02662-1

**Published:** 2026-04-16

**Authors:** Hongquan Wang, Joshua S. Fleishman, Sihang Cheng, Weixue Wang, Fan Wu, Yumin Wang, Yu Wang

**Affiliations:** 1 Department of Geriatrics, Aerospace Center Hospital, Peking University Aerospace School of Clinical Medicine, Beijing, 100049 China; 2 Department of Pharmaceutical Sciences, College of Pharmacy and Health Sciences, St. John’s University, Queens, NY 11439 USA; 3 Department of Radiology, Peking Union Medical College Hospital, Chinese Academy of Medical Sciences, Beijing, 100730 China; 4 Department of Hepatobiliary Surgery, National Clinical Research Center for Cancer/Cancer Hospital, National Cancer Center, Chinese Academy of Medical Sciences and Peking Union Medical College, Beijing, China; 5 Department of Respiratory and Critical Care Medicine, Aerospace Center Hospital, Peking University Aerospace School of Clinical Medicine, Beijing, 100049 China

**Retraction Note: Molecular Cancer 23, 177 (2024)**

**https://doi.org/10.1186/s12943-024-02088-7**

The Editor-in-Chief has retracted this article because it shows significant overlap with [1], which was under consideration at the same time and shares one author in common.

Hongquan Wang and Yumin Wang disagree with this retraction. None of the remaining authors have responded to correspondence from the Publisher about this retraction.
